# Changing Trends in Computational Drug Repositioning

**DOI:** 10.3390/ph11020057

**Published:** 2018-06-05

**Authors:** Jaswanth K. Yella, Suryanarayana Yaddanapudi, Yunguan Wang, Anil G. Jegga

**Affiliations:** 1 Division of Biomedical Informatics, Cincinnati Children’s Hospital Medical Center, 240 Albert Sabin Way MLC 7024, Cincinnati, OH 45229, USA; Jaswanth.Yella@cchmc.org (J.K.Y.); Suryanarayana.Yaddanapudi@cchmc.org (S.Y.); Yunguan.Wang@cchmc.org (Y.W.); 2 Department of Pediatrics, University of Cincinnati College of Medicine, Cincinnati, OH 45267, USA; 3 Department of Computer Science, University of Cincinnati College of Engineering, Cincinnati, OH 45219, USA

**Keywords:** computational drug repositioning, drug repositioning, drug repurposing, machine learning, deep learning, crowdsourcing, open innovation, drug discovery

## Abstract

Efforts to maximize the indications potential and revenue from drugs that are already marketed are largely motivated by what Sir James Black, a Nobel Prize-winning pharmacologist advocated—“The most fruitful basis for the discovery of a new drug is to start with an old drug”. However, rational design of drug mixtures poses formidable challenges because of the lack of or limited information about in vivo cell regulation, mechanisms of genetic pathway activation, and in vivo pathway interactions. Hence, most of the successfully repositioned drugs are the result of “serendipity”, discovered during late phase clinical studies of unexpected but beneficial findings. The connections between drug candidates and their potential adverse drug reactions or new applications are often difficult to foresee because the underlying mechanism associating them is largely unknown, complex, or dispersed and buried in silos of information. Discovery of such multi-domain *pharmacomodules*—pharmacologically relevant sub-networks of biomolecules and/or pathways—from collection of databases by independent/simultaneous mining of multiple datasets is an active area of research. Here, while presenting some of the promising bioinformatics approaches and pipelines, we summarize and discuss the current and evolving landscape of computational drug repositioning.

## 1. Introduction

The path to new drug discovery has always been a road full of twists and turns. De novo drug discovery in particular is an expensive, time-consuming, and high risk process. For instance, the total average cost of developing a new drug, as per an estimate, ranges from $2 billion to $3 billion and it takes at least 13–15 years to bring a drug to the market—starting from initial discovery to the approval stage [1]. Further, the process suffers from a high rate of attrition. About 10% of the drugs that enter into clinical trials get approved by regulatory agencies [2]. The remaining 90% of the drugs fail due to inefficacy or high toxicity due to the limited predictive value of preclinical studies [3]. Nearly 62% of the compounds fail in Phase II and approximately 45% attrition occurs in Phase III [4]. These attritions are due to insufficient R&D productivity in identifying the drug response on the target due to the limited availability of preclinical disease models which has raised concerns in the pharmaceutical industry [5]. Despite rapid technological advances and exponential increases in pharmaceutical R&D investments, the number of newly approved drugs continues to be the same [6]. To overcome these challenges and to potentially bypass this productivity gap, more and more companies are resorting to “drug repositioning” or “drug repurposing” (sometimes also referred to as drug reprofiling, drug retasking, or therapeutic switching) or simply identifying and developing new therapeutic uses for existing or abandoned pharmacotherapies [7]. The premise is that since most approved compounds have known bioavailability and safety profiles, proven formulation and manufacturing routes, and reasonably characterized pharmacology, repositioned drugs can enter clinical phases more rapidly and at a lower cost than novel compounds. Further, the 90% therapeutic development failure rate means there are many existing, partially developed therapeutic candidates that could be re-visited, explored further, and potentially repurposed for a new disease, common or rare. It is therefore not surprising that in recent years, of the new drugs that reach their first markets, repositioned drugs have taken up to a percentage of ~30%! For instance, of the 113 new drugs and biologics approved or launched in 2017, only seven were first-in-class agents (an approved and launched first drug with a novel mechanism of action) while 36 were repositioned drugs [8]. As per an estimate, this bypassing can potentially make a drug available for use in patients within 3–12 years with a total estimated cost of $40–80 million [9,10].

Most of the successful cases of drug repurposing have been serendipitous discoveries rather than systematic, hypothesis-driven outcomes. These include the accidental discovery of thalidomide as an agent for leprosy or the more notable example of sildenafil, an angina medication developed in 1989 subsequently marketed as Viagra^®^, a blockbuster drug to treat erectile dysfunction [12] (see Table 1 for additional examples of drug repositioning). De novo drug therapies for more than 8000 orphan or rare diseases are impossible to develop with the current R&D costs, however, drug repositioning with its premise of discovering hidden connections or building connections between a drug and disease hold promise for orphan disease therapy [13]. Further, revisiting the approved drugs for identifying new indications helps the pharmaceutical companies to extend the patent life of drugs, through application to adjacent diseases and also helps the company to protect the IP against competitors [14].

In-silico methods like data-mining, machine learning, and network-based approaches, offer an unprecedented opportunity to predict all possible drug repositioning candidates using available diverse and heterogeneous data sources from genomics and biomedical domains [15]. Indeed, predictive models have been built using these methods exploiting existing data such as protein targets, chemical structure, or phenotypic information such as profiles of side-effect, gene expression, etc. While the advances in computational sciences bring the possibility of applying novel algorithms and approaches to systems biology data, these datasets themselves have triggered fundamental research on more complex problems [16]. As a result of this hybrid approach of utilizing computational methods and experimental screenings, various modalities of drug repositioning methods have emerged. Computational drug repositioning methods focus on shared characteristics between two drugs and depending on what kind of drug discovery (drug-based or disease-based) [17], the methods can be classified in to target-based, expression-based, knowledge-based, chemical structure-based, pathway-based and mechanism of action-based [18]. In this review article, we briefly outline the recent progress in computational methods and strategies applied on the drug-disease data for drug repositioning investigations.

## 2. Approaches

In silico drug repurposing challenges that are drug-centric (i.e., discovering new indications for existing drugs) or disease-centric (i.e., identifying an effective drug as a potential treatment for disease) have the common challenge of either assessing the similarity or connections between drugs or between diseases [19]. Jin and Wong [18] reviewed a variety of approaches used as a basis for computational drug repurposing. These can be broadly categorized as knowledge-based and signature-based approaches.

### 2.1. Knowledge-Based Drug Repurposing

This repurposing method utilizes the available information on drug such as drug-targets, chemical structures, adverse effects, pathways etc. and builds computational models to predict unknown mechanisms, targets or new bio-markers for diseases [20,21,22,23,24]. In pathway-based approach, signaling pathways, metabolic pathways and protein-interaction networks data are used to compute the similarity or connections between drug and disease. The processed omics data, for example, from human patients or animal models of disease are used to reconstruct disease-specific pathways that can serve as key targets for novel therapeutic discovery or for repositioned drugs [25,26,27,28,29,30]. Target mechanism-based approaches on the other hand take into account known mechanism of action and target role : Here, the data available on signaling pathways, protein interactions and omics data are integrated to identify the potential mechanism of action (MoA) of drugs [31,32,33,34]. This in turn can enable find better and even specific drug targets and also for discover of an alternate medication for any disease.

### 2.2. Signature-Based Drug Repurposing

This method makes use of gene expression signatures by comparing drug gene expression profiles and disease gene expression profiles and is frequently referred to as ‘signature reversion’ method [35]. Gene expression based methods are effective in constructing a detailed map of connections between diseases and drug actions [36,37,38,39,40].

Connectivity Map (CMap) [83,84], NCBI’s Gene Expression (GEO) [75], and the relatively recent LINCS datasets [44] are also extensively explored in drug repositioning studies. Recent technical and technological advancements in molecular biology and exponential growth of biomedical data while presenting challenges have also opened up an array of opportunities to develop and apply novel and powerful computational approaches that can enable informed drug repositioning. The free availability of data repositories are further directing and catalyzing these efforts. In Table 2 we present some of the widely used open source drug- and disease-centric and related databases. These include, for instance, databases that provide information on the known targets, mechanism of action, gene expression, clinical status, ADMET properties, signaling pathways and disease-centric database which has omics data (transcriptomic, proteomic, genetic characteristics of diseases).

### 2.3. In Silico Methods for Drug Repositioning

In the following sections, we present an overview of some of the in silico methods—current and emerging—used for facilitating drug repositioning candidate discovery.

#### 2.3.1. Machine Learning

Any machine learning workflow typically comprises of 4 steps: data pre-processing, feature extraction, model fitting and evaluation [85]. PREDICT, is a similarity based machine learning framework, integrating drug-drug similarity (based on drug-protein interactions, sequence and gene-ontology) and disease-disease similarity (disease-phenotype and human phenotype ontology) where the authors have used them as features applying logistic regression to predict similar drugs for similar diseases and they achieved AUC = 0.9 in predicting drug indications [86]. SPACE, another similarity-based method predicts anatomical therapeutic chemical classification of drugs by integrating multiple data sources using Logistic Regression [87]. Likewise, several such similarity based methods have been reported for predicting novel drug indications [88,89,90].

Deep learning, a large class of machine learning-based models composed of multiple processing layers representing data with a high level of abstraction are now being explored computational biology field for a wide-variety of applications including drug discovery [91,92]. The principal difference between conventional “shallow” learning (neural network with one or two hidden layers) and deep learning is that while the former does not deal with raw data and requires a feature extraction step to be performed before the learning process, the latter not only discovers intricate structure in large data sets but by using the backpropagation algorithm allows changing the internal parameters incrementally to compute the representation in each layer from the representation in the previous layer [92]. Deep learning-based approaches have dramatically improved the state-of-the-art in speech recognition, visual object recognition, object detection and are currently being explored in biomedical and genomic domains. Aliper and Plis, for example, used deep learning with gene expression data to learn drug therapeutic categories and found that deep neural networks surpassed SVM after 10 fold cross validation suggesting a working proof for applying deep learning for drug discovery and development [93]. Interestingly, Zhao and Cheong, compared deep neural networks (DNN) approach with SVM-based approach to predict psychiatric drug indications based on the expression profiles of drugs and reported that [37]. While more studies are needed to understand if DNN-based approaches indeed have the claimed benefits, there have been additional reports suggesting that deep learning-based approaches perform better than traditional machine learning algorithms in toxicity prediction by enabling multi-task learning [94,95].

#### 2.3.2. Network Models

Network-based approaches have been extensively exploited in computational drug repositioning for identifying novel drug targets, interactions, and indications [96]. Typically, in these models, the nodes in the networks represent either drug, disease, or gene products and edges represent the interactions or relationships between them. These networks are either knowledge-based or computationally inferred using multiple data resources and have various representations such as drug-drug, drug-target, drug-disease, disease-disease, disease-gene, disease-drug, protein-protein interactions, and transcriptional networks [97]. Cheng and Liu computed similarities—drug-based, target-based, and network-based—to predict drug-target interaction in a bi-partite network and found that network based inference method performed best with an average ROC AUC of 0.96 [21]. Similar homogenous or bipartite network models have been incorporated using phenotype data such as side-effect [98,99,100], transcriptional [101,102,103], drug-disease [104,105] and signaling pathway data [25].

Integrating heterogeneous data also provides diverse information and has the potential to unveil hidden or unknown drug-disease relationships based on the guilt-by-association principle. Most of the similarity-based methods are either drug-centric or disease-centric networks, with relatively few approaches that built a drug-disease heterogeneous network using compendia of gene annotations and network clustering to identify drug repositioning candidates [105,106]. Luo and Zhao, built a similar network-based framework using heterogeneous data through a network diffusion process and used the diffusion distributions to derive the prediction scores of drug-target interactions [107]. Recently, Himmelstein et al. integrated data from 29 public resources to identify dug repositioning candidates and predicted the probability of repositioning for 209,168 drug-disease pairs [108].

#### 2.3.3. Mining Electronic Health Records for Drug Repurposing

Electronic health records (EHR) of the patients which provide medications details along with patient history can also be mined to identify drug repositioning candidates. Applying natural language processing on EHRs, for instance, reveals post-market, additional adverse drug events which are not found in clinical trials [109]. These side-effects information can be potentially used for drug-repositioning and validation [23]. Mining EHR records for example helped in identifying that metformin, a most commonly prescribed medication for type II diabetes, can also be repurposed for cancer treatment [110]. The relevance and accuracy of the model’s prediction needs to be assessed in discovering a drug whose indications are unknown. The validity of novel drug prediction can be evaluated by comparing the predicted targets in ClinicalTrials.gov, PubMed abstracts or EHR records. The performance of the model can be evaluated by computing area under the ROC curve (AUC ROC) and Precision Recall (PR) curve. Sensitivity is a metric to measure the proportion of true positive identified correctly and Specificity is the proportion of negatives correctly identified as negatives. Due to the large unannotated drug-indication pairs as false positives, the sensitivity and specificity estimates are poor and creates substantial imbalance of true positives and true negatives. In a recent review, Brown and Patel suggest that using sensitivity-validation alone is ideal since it does not need the true negatives. The authors further suggest that investigators should test their model performance with cross-validation to prevent over-fitting and weak predictive performance [111].

### 2.4. Open Innovation—Crowd Sourcing

Crowd sourcing is a collaborative approach of delegating tasks to the crowd where the variety of expertise available generates new insights or hypothesis with the available data. This paradigm has been taken advantage in a multitude areas from diverse domains including health care and genomics. The open source drug discovery process enables faster translation of research to results with a clear definition on specific problem, task decomposition and immediate feedback loop [112,113,114,115]. Pharmaceutical companies, due to the limitations in R&D business model and man power often are focused on specific diseases which may or may not include rare and neglected diseases. Hence, few pharmaceutical and non-profit companies have used crowdsourcing platforms and embraced a wide-variety of innovative solutions [116,117] directing towards discussing the scientific enigmas. Several open innovation platforms have been established in order to build industry-academia partnerships and to explore science and business opportunities with mutual benefit (Table 3).

#### National Center for Advancing Translational Sciences (NCATS)—NIH-Academia-Industry Partnerships Initiative

The National Institutes of Health (NIH), as part of the new therapeutic uses program, launched (i) NCATS’ NIH-Industry Partnerships initiative in 2012 to foster collaboration between pharmaceutical companies and the biomedical research community; and (ii) bench-to clinical repurposing initiative to test the utility of crowdsourcing efforts or computational approaches for drug repurposing.

The focus of the *match-making* NIH-industry partnerships projects is to match researchers with *open assets* from pharmaceutical assets to fuel and accelerate drug repurposing candidate discovery. Through this initiative, NCATS supports and advances research on a wide range of common and rare (including neglected) diseases. Current industry partners in this initiative include: AstraZeneca, AbbVie, Bristol-Myers Squibb, Eli Lilly, GlaxoSmithKline, Janssen Pharmaceuticals, MedImmune, Mereo BioPharma, Pfizer, and Sanofi. The participating companies make a number of partially developed assets available to academic researchers to crowdsource repurposing ideas. Projects using most of these assets can go directly into Phase II clinical trials, while some may require additional pre-clinical investigations or a Phase I clinical trial (e.g., testing in target populations to determine dosing, assess safety and tolerability).

Through the bench-to-clinic repurposing program, NCATS supports pre-clinical studies, clinical feasibility studies or proof-of-concept clinical trials to assess the utility of computational approaches or crowdsourcing efforts in discovering drug repurposing candidates. Table 4 lists the new therapeutic uses projects funded by NIH-NCATS through these two programs (additional details can be found at https://ncats.nih.gov/ntu/projects).

### 2.5. Open Source Software

The open source movement has created a substantial value in pursuing towards “*state-of-the-art*” research over the last decade with the help of reusable and generic software libraries for data processing [124,125]. Jupyter Notebook for instance is the modern data analysis tool for reproducible computational research that supports open source languages like Python, Julia, C++, R and several other languages and provides rich features for interactive computing, visualization, and documentation [126]. Structured data tools like Scikit-learn [127], R-Programming, Orange [128] and Weka [129] are useful for mining, analysis, learning and statistical computing. For the high dimensional un-structured data such as images, text, or audio outputs, deep learning tools like TensorFlow [130], Keras [131], PyTorch [132], CNTK [133], and Matlab [134] that take advantage of multi-GPU accelerated training are increasingly used. Gephi [135] and Cytoscape [136] are other popular tools used primarily for bimolecular interaction networks, omics-data integration, clustering and visualization. In Table 5, we summarize few such used tools used in computational drug discovery and repositioning.

## 3. Discussion

Drug repositioning acts as a viable strategy for a cost-effective de novo drug discovery. Although in silico methods have proven to be successful in addressing the problem of repurposing, some challenges continue to be addressed. One of the principal issues is the missing drug-disease indication data. Marking the missing indications as true negatives or ignoring them from training can potentially compromise the predictive power of the computational model for drug repurposing candidate discovery. Second, the lack of a true gold standard dataset for drug repositioning makes it difficult for in silico methods to evaluate results. As a result, common performance metrics such as sensitivity, specificity, and precision are used to assess the utility of computational drug repurposing algorithms. Third, existing computational methods tend to be predominantly one-sided (e.g., drug-centric or disease-centric). However, the integration of multi-omic data with similarity measures have been shown to have better predictive performance with identification of novel therapeutic compounds [105,108].

The sea of biomedical information (see Table 2), in which small molecule and gene/protein structural, functional and process knowledge—both in normal and disease states—is embedded consists of unstructured free-text as in publications and structured or semi-structured relational databases. Transforming information from these silos into actionable knowledge is facilitated by establishing connectivity among the subsets taken from these multiple heterogeneous and diverse domains. For example, a *pharmacomodule* consisting of a group of genes, biological processes, pathways, phenotypes, small molecules (approved drugs or investigational compounds), and a group of drug-induced or related adverse events forms a meaningful multi-domain module when the interdependency among most of the pairs of subsets are supported by scientific evidence (literature or databases). These *pharmacomodules* can potentially take us closer to answering the *how* question about the underlying a hypothetical mechanism of action or phenomena. An informed answer to the *how* question holds the premise to generate better and informed drug repositioning hypotheses. Growing scientific evidence [7] suggests that any compound found to be safe in humans is likely to have multiple therapeutic uses. However, almost all successful drug reposition crossovers so far have been the result of either accidental occurrences or informed guesses. Given that this “back-to-basics” approach for repositioning is growing in popularity [8], there is an urgent need for more efficient and systematic computational approaches to first systematize the available genomic and pharmacological databases for representation and knowledge discovery and then use these databases and pattern discovery tools to identify the potential new uses for existing drugs. What is needed clearly is a paradigm shift in the approaches—genomic, biopharmacological, and computational—for a more informed systematic drug rediscovery (“systematic serendipity”) taking into account all of the data resources. Originally coined by Eugene Garfield, “systematic serendipity” refers to the organized process of discovering previously unknown scientific relations using citation databases, leading to better possibilities for a collaboration of human serendipity with computer supported knowledge discovery [150].

The credibility of published research will improve the discoveries in science if the provided compendium has an evidence for the accuracy and reproducibility of the results. Reproducibility particularly is a major issue especially when scientific papers publish unexpected, positive results and other researchers or an independent research group is unable to replicate the same results even after using same or similar methods as reported by the original study [151,152]. It has been estimated that the irreproducible research costs up to $28 billion per year [153]! Providing the code and data used to obtain the claimed and reported results is always a better strategy than mere describing them in natural language in the paper and can be eventually an incremental step towards a better science [154,155,156]. The recent Findability, Accessibility, Interoperability, Reusability (FAIR) data principles go beyond the mere reuse of data by individuals but rather enhance the ability of machines to support and find and use the data automatically. These include any efforts that support discovery and reproducibility through good data management practices such as good data management, maintenance of the data flow, and sharing relevant tools or pipelines used in the research [157]. The recent Datasets2Tools project is in compliance with these principles and enables users to search for contributed canned analyses, datasets and tools [158]. Computational science research can be replicated effectively using tools like code version control software like Github [159] and transferable computational environments like Docker [160]. Over the past few years, the reproducibility issue is being taken seriously and many journals insist on providing code and data when submitting the paper.

In summary, emerging and advanced novel computational methods and crowdsourcing-based approaches that enable the joint analysis of genomic, biomedical and pharmacological data hold the premise to facilitate informed, efficient, and systematic drug repositioning. Whether this premise expedites drug development pipelines and how much of it translates into novel therapeutic discovery and impacts public health, especially catering to unmet needs (e.g., rare and neglected diseases), positively remains to be seen.

## Figures and Tables

**Table 1:** Examples of repositioned drugs (adapted in part from [11], this list is neither extensive nor exhaustive).

Drug	Original Indication	New Indication
Allopurinol	Cancer	Gout
Amantadine	Influenza	Parkinson’s disease
Amphotericin	Antifungal	Leishmaniasis
Arsenic	Syphilis	Leukemia
Aspirin	Inflammation, pain	Antiplatelet
Atomexetine	Depressive disorder	ADHD
Bimatoprost	Glaucoma	Promoting eyelash growth
Bromocriptine	Parkinson’s disease	Diabetes mellitus
Bupropion	Depression	Smoking cessation
Colchicine	Gout	Recurrent pericarditis
Colesevelam	Hyperlipidemia	Type 2 diabetes mellitus
Dapsone	Leprosy	Malaria
Disulfiram	Alcoholism	Melanoma
Doxepin	Depressive disorder	Antipruritic
Eflornithine	Depression	ADHD
Finasteride	Benign prostatic hyperplasia	Male pattern baldness
Gabapentin	Epilepsy	Neuropathic pain
Gemcitabine	Antiviral	Cancer
Lomitapide	Lipidemia	Familial hypercholesterolemia
Methotrexate	Cancer	Psoriasis, rheumatoid arthritis
Miltefosine	Cancer	Visceral leishmaniasis
Minoxidil	Hypertension	Hair loss
Naltrexone	Opioid addiction	Alcohol withdrawal
Naproxen	Inflammation, pain	Alzheimer’s disease
Nortriptyline	Depression	Neuropathic pain
Premetrexed	Mesothelioma	Lung cancer
Propranolol	Hypertension	Migraine prophylaxis
Raloxifene	Contraceptive	Osteoporosis
Sildenafil	Angina	Erectile dysfunction; pulmonary hypertension
Thalidomide	Morning sickness	Leprosy; multiple myeloma
Tretinoin	Acne	Leukemia
Zidovudine	Cancer	HIV/AIDS
Zileuton	Asthma	Acne

**Table 2:** Drug and Disease Centric Database Resources.

Database	Type	Description	URL	Ref.
ADReCS	Drug	System Toxicology and in silico drug safety evaluation. Contains 137,619 Drug-ADR pairs	http://bioinf.xmu.edu.cn/ADReCS/	[41]
ChEMBL	Drug	Database of bioactive drug-like small molecules and abstracted bioactivities	https://www.ebi.ac.uk/chembl	[42]
ChemSpider	Drug	Database of 64 million chemical structures	http://www.chemspider.com/	[43]
Clue (L1000 Platform)	Drug	Dataset of transcriptional responses of human cells to chemical and genetic perturbation. 1.2 Million L1000 profiles and tools for their analysis.	https://clue.io/	[44]
Comparative Toxicogenomics Database	Drug	Associations of Drug-Gene, Gene-Disease, Drug-Disease and gene-gene	http://ctdbase.org/	[45]
DailyMED	Drug	Catalogue of drug listings/drug label information	https://dailymed.nlm.nih.gov/dailymed/	[46]
DGIdb	Drug	Drug-gene annotations, interactions and potential drug ability database	http://dgidb.org/	[47]
DrugBank	Drug	Contains 11,000 drug entries and each entry contains more than 200 data fields of chemical information and drug targets.	https://www.drugbank.ca/	[48]
DrugCentral	Drug	Information on active ingredients chemical entities, pharmaceutical products, drug mode of action, indications, pharmacologic action	http://drugcentral.org/	[49]
e-Drug3D	Drug	e-Drug3D offers a facility to explore FDA approved drugs and active metabolites	http://chemoinfo.ipmc.cnrs.fr/MOLDB/index.html	[50]
Genomics of Drug Sensitivity in Cancer (GDSC)	Drug	Screenings of >1000 genetically characterized human cancer cell lines with a wide range of anti-cancer therapeutics	http://www.cancerrxgene.org/	[51]
Inxight Drugs	Drug	A comprehensive portal for drug development information from NCATS	https://drugs.ncats.io/ginas/app	
Open Targets Platform	Drug	comprehensive and robust data integration for access to and visualization of potential drug targets associated with disease	https://www.targetvalidation.org	[52]
PharmGKB	Drug	Curated dataset of genetic variation on drug response	https://www.pharmgkb.org/	[53]
pkCSM	Drug	Small-molecule pharmacokinetic (ADMET) properties prediction using SMILE data	http://biosig.unimelb.edu.au/pkcsm/prediction	[54]
Project Achilles	Drug	A genome-wide catalog of tumor dependencies, to identify vulnerabilities associated with genetic and epigenetic alterations	https://portals.broadinstitute.org/achilles	[55]
Promiscuous	Drug	Database contains three different types of entities: drugs, proteins and side-effects as well as relations between them	http://bioinformatics.charite.de/promiscuous/	[56]
PubChem	Drug	PubChem contains more than 90 million compounds chemical information along with their bio activities, gene and protein targets	http://pubchem.ncbi.nlm.nih.gov/	[57]
SIDER	Drug	Information on marketed medicines and their recorded adverse drug reactions	http://sideeffects.embl.de/	[58]
STITCH	Drug	68,000 chemicals, interactions and over 1.5 million proteins in 373 species	http://stitch.embl.de/	[59]
SuperPred	Drug	A prediction webserver for ATC code and target prediction of compounds	http://prediction.charite.de/	[60]
Therapeutic Target Database (TTD)	Drug	Dataset of known and explored therapeutic protein and nucleic acid targets, the targeted disease, pathway information and the corresponding drugs directed at each of these target	http://bidd.nus.edu.sg/group/cjttd/	[61]
Toxin and Toxin-Target Database (T3DB)	Drug	A database of 3673 toxins described by 41,733 synonyms, including pollutants, pesticides, drugs, and food toxins, which are linked to 2087 corresponding toxin target records	http://www.t3db.ca/	[62]
Human Protein Atlas	Disease and Drug	Consists of three separate parts; the Tissue Atlas showing the distribution of the proteins across all major tissues and organs in the human body, the Cell Atlas showing the subcellular localization of proteins in single cells, and finally the Pathology Atlas showing the impact of protein levels for survival of patients with cancer.	https://www.proteinatlas.org/	[63]
KEGG Medicus	Disease and Drug	Collection of databases dealing with genomes, biological pathways, diseases, drugs, and chemical substances	http://www.genome.jp/kegg/disease/ http://www.kegg.jp/ http://www.genome.jp/kegg/drug/	[64]
PsychEncode	Disease		https://www.synapse.org//#!Synapse:syn4921369/wiki/235539	[65]
Allen Brain Atlas	Disease	Gene expression maps for mouse and human brain	http://www.brain-map.org/	[66]
ArrayExpress	Disease	Micro array gene expression data at EBI	https://www.ebi.ac.uk/arrayexpress	[67]
CCLE	Disease	Database of mRNA expression and mutation data over 1100 cancer cell lines	https://portals.broadinstitute.org/ccle	[68]
COSMIC	Disease	Catalogue of somatic mutations in human cancer	http://cancer.sanger.ac.uk/cosmic	[69]
dbGAP	Disease	Catalogue of somatic mutations causing cancer	http://www.ncbi.nlm.nih.gov/gap	[70]
dbSNP	Disease	Database of single nucleotide polymorphisms	https://www.ncbi.nlm.nih.gov/snp	[71]
dbVar	Disease	Public archives for genomic structural variation	https://www.ncbi.nlm.nih.gov/dbvar	[72]
DisGeNET	Disease	Database on human disease-associated genes and variants	http://www.disgenet.org/	[73]
ENCODE	Disease	Database of comprehensive parts list of functional elements in human genome	https://genome.ucsc.edu/ENCODE/	[20]
Genomics Data Commons	Disease	Harmonized Cancer Datasets with 40 cancer mutated gene projects, 22,147 Genes and 3 million mutations	https://gdc.cancer.gov/	[74]
GEO	Disease	High throughput gene expression datasets	http://www.ncbi.nlm.nih.gov/geo	[75]
GTex	Disease	Catalog of genetic variations and their influence on gene expressions	https://www.gtexportal.org/home/	[76]
Human Proteome Map	Disease	Interactive resource with massive peptide sequencing results	http://www.humanproteomemap.org/	[77]
ICGC	Disease	Dataset with more than 17,000 cancer donors spanning 76 projects and 21 tumor sites	http://icgc.org/	[78]
IGSR	Disease	1000 genome project data usability and extension	http://www.internationalgenome.org/	[79]
Orphadata	Disease	Rare diseases, drugs and associated genes	http://www.orphadata.org/cgi-bin/index.php/	[80]
Roadmap Epigenomics	Disease	Epigenomic maps for stem cells and primary ex vivo tissues selected to represent the normal counterparts of tissues and organ systems frequently involved in human disease	http://www.roadmapepigenomics.org/	[81]
STRING	Disease	Protein-Protein interaction, analysis, and networks	https://string-db.org/cgi/input.pl	[82]

**Table 3:** Open innovation research resources.

Name	Description	URL
Centers for Therapeutic Innovation (CTI)	Collaborative research platform for clinical applications and drug discovery [118]	https://www.pfizercti.com
CREEDS	Crowd-extracted expression of differential signatures [119]	http://amp.pharm.mssm.edu/CREEDS
Grants4Leads	Financial support for exploration of new approaches in infectious diseases [120]	https://www.grants4leads.com/
Kaggle	Data scientists and statisticians competition platform with few bioinformatics challenges [117,121]	http://www.kaggle.com/
Open Innovation Drug Discovery	Academic and Industry researchers open collaboration platform for drug discovery [122]	https://openinnovation.lilly.com/dd/
Sage Bionetworks	Bioinformatics and data science challenge platform building prognostic models for breast cancer [123]	http://sagebionetworks.org/
TopCoder	Machine learning engineers, programmers and data scientists challenge platform [116]	http://www.topcoder.com

**Table 4:** NIH-NCATS funded new therapeutic uses projects (2013–2018).

Project/Study Title	Year	NCATS Program	Condition
The Efficacy and Safety of a Selective Estrogen Receptor Beta Agonist (LY500307)	2013	NIH-Industry Partnership	Schizophrenia
Fyn Inhibition by AZD0530 for Alzheimer’s Disease	2013	NIH-Industry Partnership	Alzheimer’s disease
Medication Development of a Novel Therapeutic for Smoking Cessation	2013	NIH-Industry Partnership	Cigarette smoking
A Novel Compound for Alcoholism Treatment: A Translational Strategy	2013	NIH-Industry Partnership	Alcoholism
Partnering to Treat an Orphan Disease: Duchenne Muscular Dystrophy	2013	NIH-Industry Partnership	Duchenne muscular dystrophy
Reuse of ZD4054 for Patients with Symptomatic Peripheral Artery Disease	2013	NIH-Industry Partnership	Peripheral artery disease
Therapeutic Strategy for Lymphangioleiomyomatosis	2013	NIH-Industry Partnership	Lymphangioleiomyomatosis
Therapeutic Strategy to Slow Progression of Calcific Aortic Valve Stenosis	2013	NIH-Industry Partnership	Calcific aortic valve stenosis
Translational Neuroscience Optimization of GlyT1 Inhibitor	2013	NIH-Industry Partnership	Schizophrenia
Anti-inflammatory Small Drug as Adjunctive Therapy to Improve Glucometabolic Variables in Obese, Insulin-Resistant Type 2 Diabetic Patients	2015	NIH-Industry Partnership	Insulin-resistant type 2 diabetes
Evaluation of AZD9291 in Glioblastoma Patients with Activated EGFR	2015	NIH-Industry Partnership	Glioblastoma
Evaluation of a Cathepsin S Inhibitor as a Potential Drug for Chagas Disease	2015	NIH-Industry Partnership	Chagas disease
Wee1 and HDAC Inhibition in Relapsed/Refractory AML	2015	NIH-Industry Partnership	Relapsed/refractory AML
Anti-Virulence Drug Repurposing Using Structural Systems Pharmacology	2016	Bench-to-Clinic	Bacterial virulence
CXCR2 Antagonism in the Immunometabolic Regulation of Type 2 Diabetes	2016	Bench-to-Clinic	Type 2 diabetes
Drug Repositioning in Diabetic Nephropathy	2016	Bench-to-Clinic	Diabetic nephropathy
Ketorolac and Related NSAIDs for Targeting Rho-Family GTPases in Ovarian Cancer	2016	Bench-to-Clinic	Ovarian cancer
Network-Driven Drug Repurposing Approaches to Treat Coronary Artery Disease	2016	Bench-to-Clinic	Coronary artery disease
Pre-Clinical Evaluation of a Neutrophil Elastase Inhibitor for the Treatment of Inflammatory Bowel Disease	2016	Bench-to-Clinic	Inflammatory bowel disease
Quantum Model Repurposing of Cethromycin for Liver Stage Malaria	2016	Bench-to-Clinic	Liver-stage malaria
Repurposing Lesogaberan for the Treatment of Type 1 Diabetes	2016	Bench-to-Clinic	Type 1 diabetes
Repurposing Misoprostol for Clostridium Difficile Colitis as Identified by PheWAS	2016	Bench-to-Clinic	Clostridium difficile colitis
Repurposing Pyronaridine as a Treatment for the Ebola Virus	2016	Bench-to-Clinic	Ebola virus
Therapeutic Repurposing of Benserazide for Colon Cancer	2016	Bench-to-Clinic	Colon cancer
Computational Repurposing of Chemotherapies for Pulmonary Hypertension	2017	Bench-to-Clinic	Pulmonary hypertension
Pre-Clinical Evaluation of Vorinostat in Alopecia Areata	2017	Bench-to-Clinic	Alopecia areata
Pre-Clinical Testing of a Novel Therapeutic for Nonalcoholic Steatohepatitis	2017	Bench-to-Clinic	Nonalcoholic steatohepatitis
Repurposing Pyronaridine as a Treatment for Chagas Disease	2017	Bench-to-Clinic	Chagas disease
Single-Cell-Driven Drug Repositioning Approaches to Target Inflammation in Atherosclerosis	2017	Bench-to-Clinic	Atherosclerosis
Impact of SAR152954 on Prenatal Alcohol Exposure-Induced Neurobehavioral Deficits	2017	Bench-to-Clinic	Neurobehavioral deficits
An Endoplasmic Reticulum Calcium Stabilizer for the Treatment of Wolfram Syndrome	2017	Bench-to-Clinic	Wolfram syndrome
Utilization of Phenotypic Precision Medicine to Identify Optimal Drug Combinations for the Treatment of Hepatocellular Carcinoma	2017	Bench-to-Clinic	Hepatocellular carcinoma
Targeting Glucose Metabolism for the Treatment of Hepatocellular Carcinoma	2017	Bench-to-Clinic	Hepatocellular carcinoma
Application of a Repurposed FDA Approved Drug as a Local Osteogenic Agent	2017	Bench-to-Clinic	To induce local osteogenesis
Repurposing Misoprostol to Prevent Recurrence of Clostridium Difficile Infection	2018	Bench-to-Clinic	Recurrent Clostridium difficile
AZD9668: A First in Class Disease Modifying Therapy to Treat Alpha-1 Antitrypsin Deficiency, a Genetically Linked Orphan Disease	2018	NIH-Industry Partnership	Alpha-1 antitrypsin deficiency
AZD9668 and Neutrophil Elastase Inhibition to Prevent Graft-versus-Host Disease	2018	NIH-Industry Partnership	Graft-versus-host disease
Use of the Src Family Kinase Inhibitor Saracatinib in the Treatment of Pulmonary Fibrosis	2018	NIH-Industry Partnership	Pulmonary fibrosis

**Table 5:** Web-tools and open source kits.

Tool	Description	URL	Ref
Clue	Tools for perturbagens (small molecules or genes) query, L1000 cohorts, and gene expression heatmap visualization	https://clue.io	[44]
Clue Repurposing Tool	Interactive application to access approved and pre-clinical drug annotations	https://clue.io/repurposing	[137]
COGENA	Analysis, visualizing and clustering tool for gene expression profiles	https://github.com/zhilongjia/cogena	[138]
DeepChem	Deep learning toolkit for drug discovery and cheminformatics	https://deepchem.io/	[139]
DR.PRODIS	Prediction of drug-protein interactions, side effects	http://cssb.biology.gatech.edu/repurpose	[140]
e-LEA3D	Collection of tools related to computer-aided drug design	http://chemoinfo.ipmc.cnrs.fr/	[141]
Frog2	Chemo-informatics toolkit for small compound 3D generation from 1D/2D input	http://bioserv.rpbs.univ-paris-diderot.fr/services/Frog2/	[142]
GIFT	Infer chemogenomic features from drug-target interactions.	http://bioinfo.au.tsinghua.edu.cn/software/GIFT/	[143]
GoPredict	Drug target prioritization tool for breast and ovarian cancer	http://csblcanges.fimm.fi/GOPredict/	[144]
JOELib/JOELib2	Toolkit to interconvert chemical file formats, descriptor calculation classes, and SMARTS substructure search	http://www.ra.cs.uni-tuebingen.de/software/joelib/introduction.html	[145]
ksRepo	Drug repositioning tool that utilizes gene expression drug datasets from different platforms	https://github.com/adam-sam-brown/ksRepo	[101]
L1000CDS	L1000 dataset based gene expression signature search engine	http://amp.pharm.mssm.edu/L1000CDS2/#/index	[146]
MANTRA	Prediction and analysis of mechanism of action of drugs for drug repositioning	http://mantra.tigem.it/	[147]
NFFinder	Tool to discover multiple drugs with similar drugs based on up/down regulated genes	http://nffinder.cnb.csic.es/	[102]
Open babel	Open source chemistry toolbox	http://openbabel.org/wiki/Main_Page	[148]
Open PHACTS	European funded initiative to bring together industry and academic partners for semantic integration of pharmacological data using an RDF data model	http://www.openphacts.org	[149]
